# Construction of the active aging index in Bangladesh: challenges and opportunities

**DOI:** 10.1016/j.heliyon.2022.e10922

**Published:** 2022-10-02

**Authors:** Md Aminul Haque, Sadiya Afrin

**Affiliations:** Department of Population Sciences, University of Dhaka, Room No: 4046, Arts Building, Dhaka, 1000, Bangladesh

**Keywords:** Active aging, Older population, Active aging index (AAI), Dimension, WHO Model for AAI, Bangladesh

## Abstract

**Background:**

Bangladesh is one of the fastest-growing older populations in the world. However, there has been little initiative in constructing an Active Aging Index (AAI) to monitor the quality of life of senior citizen.

**Objectives:**

The objective of the study was to construct an AAI to know the active aging scenario of the population in Bangladesh.

**Methods:**

A cross-sectional study was conducted among 518 respondents aged 60 years or older from six villages and six wards. Three villages and three wards from Rangpur district and three villages and three wards from Dhaka district were selected for the collection of data. A semi-structured questionnaire was used to collect data on the eight indicators of the ‘health dimension’, three indicators of the ‘participation dimension’, and seven indicators of the ‘security dimension’ for the AAI using the World Health Organization (WHO) model. The responses for each of the indicators (ranges from 0, 1, 2, or 3) in each dimension were added to create a composite index (CI) for each of the dimensions. Descriptive statistical methods with significance tests were applied to analyze the data.

**Results:**

The findings provide opportunities to view the 18 aspects of the quality of life of the older population by sex and place of residence. Among the respondents, the overall AAI score shows that 48.1% of the health, 28.9% of the participation, and 48.5% of the security dimensions score fall in the lower active category. Gender differences were distinct in all three dimensions, where the moderate AAI score for females from both rural (41.5%) and urban areas (62.7%) was lower than for males in rural (73.5 %) and urban areas (76.3%). Conversely, the AAI value for urban older adults was higher in all dimensions in comparison with rural older adults. Overall, 62.7% of the respondents were moderately active (a range within 0.50–0.79), while 34.4% were poorly active (a range within 0.0–0.49) and only 3.9% were highly active (a range within 0.80+).

**Conclusion:**

Effective initiatives are needed to improve the individual scores of each of the three dimensions of the AAI. Attention should be given to addressing the gender and residential variations in all three dimensions of the AAI. The incorporation of indicator-specific measures is essential to the existing plan of action and programs to improve the situation of the older population that is poorly and moderately active. This result will help policymakers from concerned ministries to focus on specific dimensions to improve the AAI situation in the country. A nationally representative study is needed regularly to monitor the AAI situation.

## 1 Introduction

The measurement of active aging has become an important research topic in recent times. This gives insight into the potential of older adults and assists decision-makers in improving policies to include them in economic and social activities and living a healthy life [1]. The future inevitable aging problem in developing countries requires proper consideration by policymakers in order to undertake sustainable aging policies [2, 3, 4, 5, 6]. There has been less attention paid to the measurement of active aging situations in developing countries [1]. Active aging is multi-dimensional and varies by the socio-economic, demographic, and health functional differentials of a population [5]. In most developing countries, including Bangladesh, older adults usually face various socio-economic vulnerabilities and deprivations [7]. The condition of older people like who are women, widows, and those living in slums and rural areas is even more aggravated [8, 9]. In Bangladesh, older adults are among the significantly deprived citizens and the situation is worse for rural older women who do not have income and access to family and health care [2, 10].

The older population is the most vulnerable group in our society because of their physical weakness, disease burden, lack of fixed income sources, and lack of property ownership [2, 8, 11, 12]. With the increasing life expectancy and economic development of the country, appropriate tools are required for measuring the needs of older adults. A better understanding of the dimensions and determinants of active aging is inevitable for a country like Bangladesh.

Similar to other developing countries, very little information is available regarding the AAI for the older adults of Bangladesh [1]. Without a proper understanding of the active aging situation, the older population of the country will suffer a lot, and the policy measures adopted by the country for the elderly will not be effective in the future. Therefore, this paper aims to calculate the AAI for Bangladesh using the WHO model based on United Nations principles for older people [15]. An AAI construction can support and contribute to policy measures that are critical for the effective management of the challenges of a rapidly growing older population in a developing country like Bangladesh. This paper outlines an introduction and review of the existing literature on AAI methods to calculate active aging, the outcomes of the AAI, which is then followed by a discussion and conclusion.

Population aging is a major global trend that affects many developing and developed countries at different rates and levels [16]. Worldwide, there are an estimated 727 million older people aged 65 or older and this number is likely to double reaching over 1.5 billion by 2050. One in every six people will be aged 65 years or over, by the middle of the twenty-first century, and population aging is a global phenomenon that requires international, national, regional, and local level action [17].

The percentage of the elderly population in Bangladesh is 7.7 percent (11.09 million) [7], which is projected to become almost 14 million in 2020 and 17.2 million in 2025 [13]. Another projection shows that the size of the elderly population is 13.0 million (8.0%) in 2019 and is expected to double to 36.0 million (21.9%) in 2050 [18]. The increasing aging population will have an impact on health status, the healthcare system, economic growth, labor markets, consumption, savings, investment, pension, and intergenerational transfers [19]. In most developing countries, demographic and epidemiological transitions are taking place in a changing socio-economic context [20, 21, 22]. Bangladesh needs to prepare to provide the necessary support for the coming surge of older adults [6, 8, 23].

Older people living in rural areas receive poor health care services and have access to fewer economic activities, and formal jobs, as opportunities are limited [8, 9]. At the same time, because of rapid urbanization in Bangladesh, a large number of elderly people will be residing in slums in the future. Without proper plans and programs for the older population, Bangladesh will face major challenges for providing support and care for the older population [2, 8]. Urbanization, higher education, and engagement in the service sector increased migration among the youth and a section of the youth are not accompanied by their parents. Situations, like the death of spouses, and living single life are common causes of loneliness in the older population [2, 8, 9]. Moreover, 95.0% of the older population has health problems including multiple health complications [3, 4].

**Figure 1:**
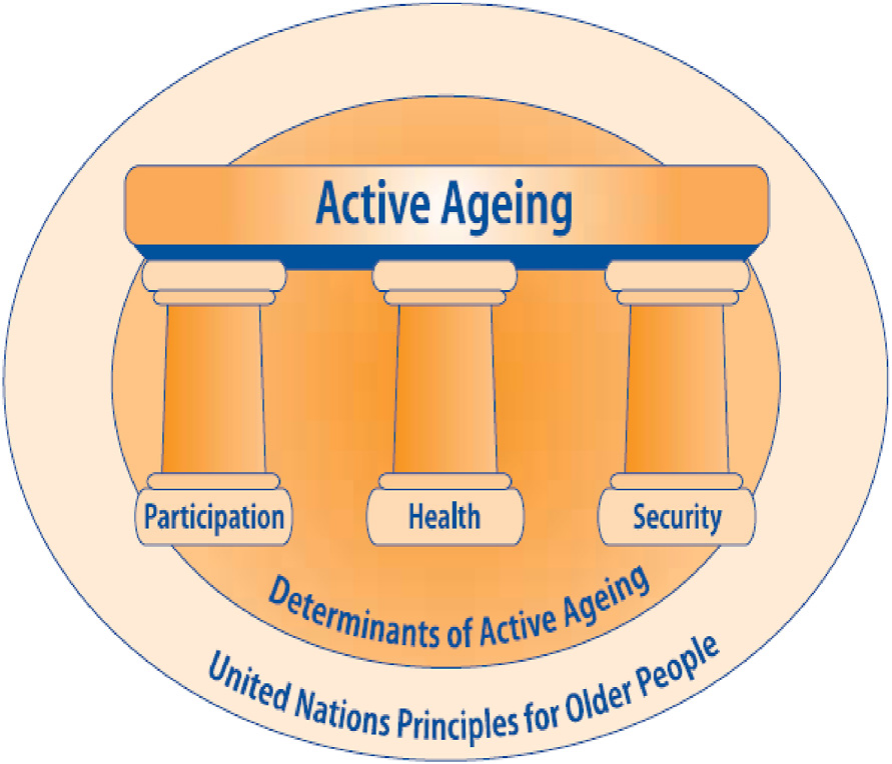
The three pillars of a policy framework for Active Aging. *Source*: (WHO, 2002).

Aging poses several challenges for individuals, societies, and governments. If the older people can be transformed into ‘active aging’ by engaging them in economic and social activities, a second dividend can be achieved. However, if a country fails to ensure involvement in economic activities, a healthy life, and social participation for the older population, then they will be a severe burden for the country [24]. The WHO has formulated a ‘policy framework’ to describe the activeness and quality of life of the older population (Figure 1). A country needs to assess the participation dimension, health dimension, and security dimension of the older population to know the overall active aging situation.

The policy framework for active aging has three main dimensions-participation, health, and security. The framework mainly focuses on issues such as participation in social and economic activities, preventing and reducing the burden of disabilities, chronic disease, premature mortality, reducing the risk factors associated with non-communicable disease, and functional decline as individuals age [15]. Considering the context of the country, availability of data, and objectives of the study, the three dimensions of the WHO model were followed [15]. However, the indicators used vary from 15 to 24. For example, 15 indicators were used in two studies from Bangladesh [25] and Thailand [5]. In other studies, 24 indicators were used in Thailand [26, 27], 22 indicators in Portugal [27], and 18 indicators in Zambia [28]. There are also two other established active aging indexes from the United Nations Economic Commission for Europe (UNECE) and HelpAge International that have different dimensions and indicators [15, 29, 30]. The UNECE model has 22 indicators under four domains to calculate AAI, and Global AgeWatch Index (GAWI) has used four domains and 13 indicators in the AgeWatch Index calculations [29, 30]. There is only one study conducted in Bangladesh using the UNECE model [31]. The Global AgeWatch index of HelpAge, published on a three-year interval, is primarily dependent on secondary open-sources data like the Human Development Index [32]. According to the ranking, globally, Bangladesh ranked 67 out of the 96 countries on the Global AgeWatch index [18]. The need for understanding the active aging situation is inevitable for a country like Bangladesh. Therefore, the study was conducted in both rural and urban areas to know the AAI situation following the WHO framework using a total of 18 indicators to find out the opportunities and challenges associated with it.

## 2 Methods

A quantitative research approach was used in this cross-sectional study. Multistage sampling strategies were used to collect data from the selected study areas. Sample size was determined using the following formula: n = Z^2^ ∗ PQ/d^2^, where, n = sample size; Z = standardized value; P = probability of the studied phenomenon; Q = 1 − P; and d = desired accuracy [33]. According to Sample Vital Registration System- 2017 [34], the proportion of older people in Bangladesh was 8.0%, so, the study adopted P = 0.8 [34], and the sampling error was 3.0%. As the study involved more than 2 stages, a design effect of 1.65 was adopted. Considering the design effect, the sample size was 518.

The respondents for this study comprised both males and females from rural and urban areas who were 60 years or older. Two from eight divisions, two districts, two upazilas, and two unions were selected randomly. Divisions to the unions are higher than lower levels of the administrative units in Bangladesh. One selected union was Birulia of Savar upazila of Dhaka district, and the other union was Raypur of Pirganj upazila of Rangpur district. All of the selected five wards had both rural and urban characteristics and one ward of Pirganj was rural. A summary of the selection of the study areas and respondents is given in Table 1. A list of the older population was prepared based on the national voter list of the concerned villages and wards. The national voter list was collected at the union level; which is the local-level administrative unit in Bangladesh. Respondents were selected randomly from the list of the older population. Based on the proportion of the older adults in rural and urban areas of the selected divisions [35], the number of rural and urban respondents in these divisions was distributed between rural (284) and urban (234) areas. To get the required number of respondents from both urban and rural areas, respondents were interviewed systematically after every two and three households from Raypur and Birulia union respectively as per the concentration of older population of these areas [35] that meet the required number of respondents as shown in Table 1.

**Table 1:** Summary of the selection of the study areas and respondent selection for the study.

Division	District	Upazila	Union	Ward
Dhaka	Dhaka	Savar	A total of 141 respondents were selected from three villages of Birulia Union	A total of 116 respondents were selected from 3 wards∗ of the Birulia paurashava area
Rangpur	Rangpur	Pirganj	A total of 143 respondents were selected from 3 villages of Raypur union	A total of 118 respondents were selected from 3 Wards of the Raypur paurashava area

∗ = smallest administrative unit of Paurashava.

A semi-structured questionnaire was prepared to focus on the indicator-specific questions of the three dimensions--health, participation, and security. A study in Thailand used the WHO policy framework and incorporated the highest number of 24 indicators. However, a few indicators of the study, such as happiness level, drinking alcohol, subjective health, annual health checkup, social support quality, and location of bed [26], did not fit with our socio-cultural reality making data collection impossible. As a result, after consulting with experts from both public and private institutions in the country, the study attempted to incorporate 18 indicators for the study. In the health dimension, there were eight questions such as self-assessed health, psychological wellbeing, disabilities, chronic illness, daily activities, functional limitations, health risk behavior, and physical exercise. In the participation domain, three questions were about family interaction, workforce participation, and club/group participation. Seven questions were asked under the security dimension that included income, source of income, sufficiency of income, house ownership, living arrangements, better toilet facilities, and safety facilities.

The Department of Population Sciences of the University of Dhaka formed a research evaluation committee, to conduct an unbiased review for approval of this study. From May to July 2019, data were collected from older population households. The respondents were informed before, and the interview schedule was set at their convenience. For each of the respondents, verbal consent was obtained beforehand starting the interview. We explained the research goals and objectives of the research to the respondents before the initiation of the interview. They had the right to stop sharing information or to not respond to any question at any time.

## 3 WHO model for AAI for this study

Health, Participation, and Security were the three main dimensions of the WHO model. For this study, 18 indicators were used to calculate the AAI under these three dimensions. The details of the dimensions, specific indicators, descriptions, and codes are presented in Table 2.

**Table 2:** Definitions and codes for the indicators under each dimension.

Indicators	Description	Code
Indicators for Health Dimension		
X_1_ = Self-assessed health status	Self-assessed health status is an individual's assessment of his or her health.	1 = poor; 2 = moderate; 3 = good
X_2_ = Psychological well-being	The perception of the sense of mental wellness in terms of self-esteem.	1 = poor; 2 = moderate; 3 = good
X_3_ = Physical disabilities	The number of handicaps such as paralysis, blindness, and deafness.	0 = 1 or more; 1 = no
X_4_ = Presence of chronic illness	Prevalence of chronic illness of older persons	0 = 2 or more, 1 = 1, 2 = none
X_5_ = Activities of daily living limitations	ADL limitations consider the inability in performing one of these three activities: eating, dressing, and bathing.	0 = 1 or more; 1 = no
X_6_ = Instrumental activities of daily living limitations	Physical limitations, such as squatting, lifting objects weighing 5 kg, walking about 1 km, and climbing stairs (2–3 steps).	0 = 1 or more; 1 = no
X_7_ = Health-risk behavior	Older person practices health-risk behavior	0 = yes; 1 = no
X_8_ = Regular walk as exercise	Older person practices regular walk as exercise	0 = no; 1 = yes
Indicators for Participation Dimension		
X_1_ = Workforce participation	Older adult still participates in paid and unpaid work	0 = no; 1 = yes
X_2_ = Family interaction	Older adults support family members, e.g. food supply, housekeeping, and childcare.	0 = no; 1 = yes
X_3_ = Clubs/groups participation	Older adult takes part in activities proposed by various groups, i.e. elderly group, religious group, voluntary social work group	0 = no; 1 = yes
Indicators for Security Dimension		
X_1_ = Income status	The income status of the older population	0 = no income; 1 = having income
X_2_ = Sufficiency of income	The self-assessment by the older persons on whether his/her income is sufficient for a living.	0 = not sufficient; 1 = sufficient
X_3_ = Source of income	The number of sources of income that an older adult receives, i.e. work, pension, government living allowance, saving/interest, spouse, children, relatives, or others.	0 = no source; 1 = 1 source; 2 = 2 or more
X_4_ = House ownership	The ownership of the dwelling in which an older person is living.	0 = no; 1 = yes
X_5_ = Living arrangements	The co-residence of older adults with family members or others in their household	0 = living alone; 1 = living with spouse/children or others
X_6_ = Better toilet facilities	Having better toilet facilities	0 = no; 1 = yes
X_7_ = Safety facilities	Suffer from fear when staying alone	0 = feel fear; 1 = feel no fear

At the first stage of the calculation of the AAI, a Composite Index (CI) for each dimension was calculated. The weighted score principle was applied for each of the indicators under the dimensions of health, participation, and security. The responses for each of the indicators in each dimension were added to create the CIs for each of the dimensions of AAI [36]. The equation for CI for each of the dimensions is shown below:

1. CI for Health Dimension: X_1_/M_1_ × T + X_2_/M_2_ × T + X_3_/M_3_ × T + X_4_/M_4_ × T + X_5_/M_5_ × T + X_6_/M_6_ × T + X_7_/M_7_ × T + X_8_/M_8_ × T
2. CI for Participation Dimension: X_1_/M_1_ × T + X_2_/M_2_ × T + X_3_/M_3_ × T
3. CI for Security Dimension: X_1_/M_1_ × T + X_2_/M_2_ × T + X_3_/M_3_ × T + X_4_/M_4_ × T + X_5_/M_5_ × T + X_6_/M_6_ × T + X_7_/M_7_ × T

Where,

X = represents the value of each indicator,

M = represents the maximum answer value for each indicator, and

T = represents the total number of indicators in each dimension.

In the second stage of AAI, Dimension Index (DI) was prepared using the calculated CI of each of the dimensions of AAI. The performance of each of the dimensions was determined as the minimum and maximum value between 0 and 1 following the Human Development Index construction method of the United Nations Development Programme [36].

The formula for the DI is given below:

$$Dimension\;Index=\frac{Actualvalue-Minimumvalue}{Maximumvalue-Minimumvalue}$$

Finally, the combined effects of the calculated DIs were used to calculate AAI. The formula for calculating AAI is given below:

AAI = 1/3 (DI for Health) + 1/3 (DI for Participation) + 1/3 (DI for Security)

Based on the human development criteria of UNDP, each index was classified into three categories:

• low = score less than 0.5
• moderate = score between 0.5 and 0.79 and
• high = score higher than 0.8.

Overall and gender and residence-specific prevalence of the active aging situations were calculated. Chi-square statistics were applied to compare the indicator and dimension-specific measurement scales (categorical variables). Data were analyzed using the SPSS software (version 22.0 for windows).

## 4 Results

Table 3 presents the dimension-specific index of the AAI by place of residence and gender. Self-assessment of the respondents about their good health shows that only 19.5 percent of the respondents have good health, 18.0 percent reported good psychological well-being, and only 30.9 percent do not have a chronic illness. Functional practice limitations are prevalent among 32.4 percent of respondents and 64.9 percent practice health risk behaviors. Males from both rural (20.4%) and urban (32.2%) areas have a higher index for good health than females from rural (9.9%) and urban (14.6%) areas. Similarly, males from both rural and urban areas have a higher score for psychological well-being than females from rural and urban.

**Table 3:** Dimension-specific indicators and their scores for constructing the active aging index.

Indicators	Measurement scale	Rural		Urban		Overall (n = 518)	Chi-square
		Male (n = 113)	Female (n = 171)	Male (n = 152)	Female (n = 82)		
**Index for Health Dimension**							
Self-assessed health status	1 = poor	54.0	68.4	23.7	42.7	48.1	33.62∗
	2 = moderate	25.7	21.6	44.1	42.7	32.4	
	3 = good	20.4	9.9	32.2	14.6	19.5	
Psychological well-being	1 = poor	56.6	70.8	34.2	42.7	52.5	23.08∗
	2 = moderate	25.7	21.1	35.5	41.5	29.5	
	3 = good	17.7	8.2	30.3	15.9	18.0	
Disabilities	0 = 1 or more	4.4	5.8	1.3	0.0	3.3	0.70
	1 = no	95.6	94.2	98.7	100	96.7	
Chronic illness	0 = 2 or more	34.5	28.7	25.7	39.0	30.7	3.05
	1 = 1	37.2	43.3	35.5	35.4	38.4	
	2 = none	28.3	28.1	38.8	25.6	30.9	
Activity of daily living (ADL) limitations	0 = 1 or more	3.5	5.3	0.7	3.7	3.3	3.33
	1 = no	96.5	94.7	99.3	96.3	96.7	
Functional limitations	0 = 1 or more	27.4	42.1	19.7	42.7	32.4	21.94∗
	1 = no	72.6	57.9	80.3	57.3	67.6	
Practice of health risk behavior	0 = 1 or more	55.8	65.5	66.4	73.2	64.9	2.11
	1 = no	44.2	34.5	3.6	26.8	35.1	
Daily Exercise	0 = no	74.3	86.0	64.5	76.8	75.7	14.43
	1 = yes	25.7	14.0	35.5	23.2	24.3	
**Index for Participation Dimension**							
Participation in workforce	0 = no	42.5	88.3	28.9	78.0	59.3	23.08∗
	1 = yes	57.5	11.7	71.1	22.0	40.7	
Interaction with family members	0 = no	8.0	11.7	5.3	4.9	7.9	0.70
	1 = yes	92.0	88.3	94.7	95.1	92.1	
Participation in group works	0 = no	54.9	71.3	65.1	69.5	65.6	3.05
	1 = yes	45.1	28.7	34.9	30.5	34.4	
**Index for Security Dimension**							
Income	0 = no	20.4	72.5	12.5	30.5	36.9	103.03∗
	1 = yes	79.6	27.5	87.5	69.5	63.1	
Sufficiency of income	0 = not sufficient	91.2	98.8	84.2	95.1	92.3	19.87∗
	1 = sufficient	8.8	1.2	15.8	4.9	7.7	
Sources of Income	0 = no	20.4	72.5	11.8	31.7	36.9	108.60∗
	1 = 1 source	42.5	17.0	51.3	24.4	33.8	
	2 = 2 or more	37.2	10.5	36.8	43.9	29.3	
House ownership	0 = no	2.7	10.5	5.9	14.6	8.1	9.33∗
	1 = yes	97.3	89.5	94.1	85.4	91.9	
Living arrangement	0 = living alone	7.1	11.7	42.8	23.2	21.6	11.24∗
	1 = with spouse, children or others	92.9	88.3	57.2	76.8	78.4	
Better Toilet facilities	0 = no	39.8	36.3	12.5	25.6	28.4	4.77∗
	1 = yes	60.2	63.7	87.5	87.4	71.6	
Safety facilities	0 = feel fear	24.8	30.6	13.8	29.3	24.2	9.59∗
	1 = feel no fear	75.2	69.4	86.2	70.3	75.8	

(∗ = significant at 0.05 level for both males and females from rural and urban areas).

Only 40.7 percent of respondents are involved in work. Participation in the work by females from both rural (11.7%) and urban (22.0%) areas is very low in comparison to male from both rural (57.5%) and urban (71.1%) areas. The results show that 36.9% of respondents have no income and 92.3% of the respondents’ incomes are insufficient among the respondents who have income (63.1%). Overall, only 7.7 percent of participants have income sufficiency, and 21.6 percent of the respondents are living alone. The results also show that 28.4 percent of respondents have poor toilet facilities, and 24.2 percent of respondents live in insecurity (feel fear) when they live alone. A chi-square test was conducted to know the significance level of the variations in the measurement scale of each of the indicators of the three dimensions. Significant variations (chi-square value) are found in 11 indicator-specific categories for both males and females (overall).

Table 4 shows that, in the health dimension, only 4.4 percent of the respondents were highly active, 47.5 percent were moderately active, and 48.1 percent were poorly active. Further, only 2.3 percent of females from rural areas and 2.4 percent of the respondents from urban areas were highly active, and only 4.0 percent of males from rural areas and 7.9 percent from urban areas were highly active.

**Table 4:** Dimension-specific composite index (CI) for active aging index.

Dimension	Description		Measurement	Rural		Urban		Overall (n = 518)	Chi-square
				Male (n = 113)	Female (n = 171)	Male (n = 152)	Female (n = 82)		
Health Index	CI constructed from eight components		1 = low	49.6	63.8	23.5	58.5	48.1	39.67∗
			2 = moderate	46.4	33.9	68.6	39.1	47.5	
			3 = high	4.0	2.3	7.9	2.4	4.4	
Participation Index	CI constructed from three components		1 = low	17.7	42.1	15.1	39.0	28.9	66.63∗
			2 = moderate	40.7	50.3	53.9	47.6	48.0	
			3 = high	41.6	7.9	30.9	13.4	22.1	
Security Index	CI constructed from seven components		1 = low	33.6	70.8	38.2	41.5	48.5	34.96∗
			2 = moderate	53.1	24.6	46.7	51.2	41.5	
			3 = high	13.3	4.6	15.3	7.3	10.0	
Overall AAI									
Overall AAI		CI constructed from three dimension	1 = low	23.9	58.5	16.4	35.0	34.4	59.98∗
			2 = moderate	73.5	41.5	76.3	62.7	62.7	
			3 = high	2.6	0.0	7.3	2.3	3.9	

(∗ = significant at 0.05 level).

The composite index for the participation dimension shows that 28.9 percent of respondents had lower participation in paid work, family interaction, and group work. Only 22.1 percent of respondents had higher participation. Higher participation percentage for males in paid work, family interaction, and group work was observed for both rural (41.6%) and urban (30.9%) areas compared to females from both rural (7.9%) and urban (13.4%) areas.

The composite index for security also shows that, 48.5 percent of respondents had lower security in income, house ownership, living arrangement, and toilet and safety facilities. Only 10.0 percent of respondents had a higher security level. The index also shows that respondents from urban areas had higher security levels than rural respondents from rural areas.

The AAI value shows that, only 3.9 percent of respondents were highly active, 62.7 percent were moderately active, and 34.4 percent were poorly active. Comparatively, high AAI for males from both rural (2.6%) and urban areas (7.3%) was seen, which is higher than for females from rural (0.0%) and urban (2.3%) areas. Calculated AAI shows that most of the male and female participants were moderately active.

**Figure 2:**
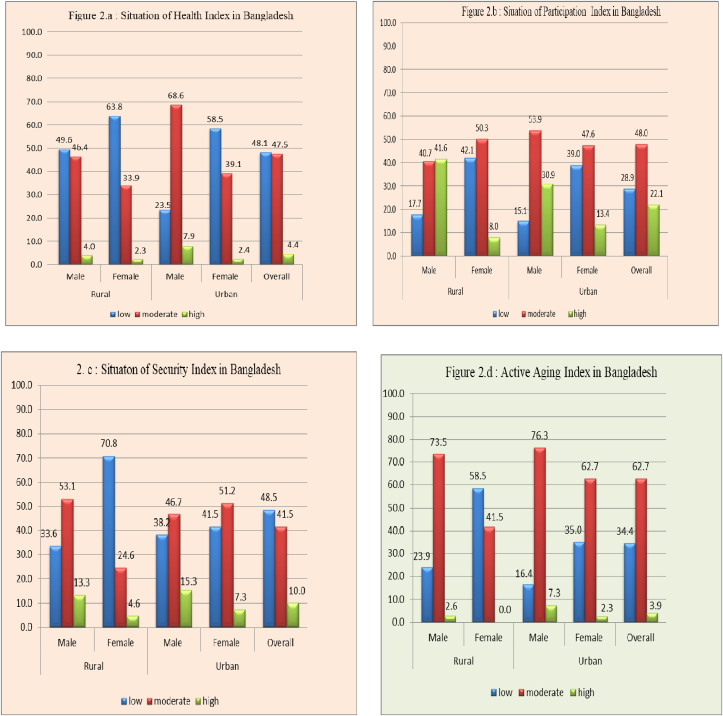
[2(a–d)]: Dimension specific Index by sex and residence and overall AAI.

Figure [2(a, b, c, d)] shows the situation of the health, participation, and security index for Bangladesh. In most cases, the index is moderate for each dimension. Females have a lower score in each dimension of the aging index. AAI for females from rural areas was very low.

## 5 Discussion

The study provides an examination of important aspects of the quality of life in the older population in a resource-poor-settings. Most of the respondents are from both rural and sub-urban settings, have a low level of formal education, and their livelihoods mainly depends on agriculture [2, 3, 6]. Still, they have quite a good understanding and have given information on the eighteen indicators used for the health, participation, and security dimensions of the proposed framework for the AAI of the WHO. The study findings inform an understanding of the quality of life in the older population. It also enables us to discern the dimension-specific scores of the AAI for the respondents. The overall health situation of the older population was poor and the prevalence of multiple chronic diseases was high (30.7%). Smoking and smokeless tobacco consumption as part of health risk behavior were highly prevalent (64.9%). Thus, efforts should be made to reduce the prevalence of chronic illness, and health risk behaviors.

Most developed countries calculate their AAI regularly and try to improve the indicators that have lower scores [31, 37]. In developing countries, few academicians and organizations have conducted the AAI for their country to bring the issue to the policymakers. The AAI using the WHO policy framework has been conducted in Sub-Saharan Africa [28, 38], Thailand [5, 26, 27], and Russia [1]. The inclusion of both males and females from rural and urban areas also added a new dimension to the existing literature. There is very little research emphasizing on the gender aspects and place of residence in constructing the AAI, especially in developing countries [1, 5, 28]. Therefore, the research findings will contribute to filling this important knowledge gap by providing information on gender aspects as well as place of residence in calculating AAI. Considering the context of the country, variations were also found in studies conducted in Sub-Saharan African countries [28, 38] and Thailand [26, 27]. Like other countries, income insecure older women are most affected in developing countries, including Bangladesh [5, 25]. The scores of the AAI vary between the elderly male and female, while women, particularly rural women, are socially disadvantaged [8, 12, 14].

Active aging-related issues have been validated in this study that covers a large number of male and female respondents and the reliability of the tools has been checked using studies in other countries [1, 5, 25, 28, 38]. A unique feature of the study is the inclusion of the high number of indicators to calculate AAI. These findings will also create an opportunity for researchers to compare with the Global AgeWatch index and the AAI of the UNECE model [18, 31]. The findings can help policy-makers identify the vulnerabilities of the elderly in terms of poor score on the indicators found in the AAI calculations. Thus, prioritized action involving the community can be taken to improve the indicators for health, participation, and security dimensions. The study findings mostly focus on the existing data gaps in the AAI for a developing country like Bangladesh, and this systematic information will allow scientists, researchers, and policymakers to design and implement strategies. The WHO model of active aging encompasses a new scope and arena for calculating and identifying the factors of active aging. This study is the first of its kind to prepare AAI using the WHO model. Some important indicators of active aging were taken into account for calculating AAI in Bangladesh, such as the existence of chronic illness, the practice of health risk behaviors, and fear in old age [15, 25, 26, 27]. Thus, the research methodology's usage of such indices and their measurements will be helpful for researchers to conduct future research on AAI.

The study reveals that the scores in the three dimensions of the AAI are lower than in other developing countries [26, 27]. More than half of older adults were moderately active in the health dimension (range between 0.50–0.79). However, 48.1 percent of older adults had a low health index (between 0–0.49). Forty-eight percent of the older adults were in the moderate ranges (0.50–0.79) of the participation index, while 28.9 percent were in the low ranges (between 0–0.49). In terms of security, 41.5 percent rated themselves as moderate (range between 0.50–0.79), while 48.5 percent rated themselves as poor (between 0–0.49). The results showed that 62.7 percent of respondents were moderately active (between 0.50–0.79), while around 34.4 percent were poorly active (range between 0–0.49). It was observed that scores for the females elderly in the AAI and on three dimensions were lower than for males. Calculated AAI values for both males and females require further improvement. The variations in rural and urban areas by sex will help researchers compare AAI in the future between developing countries with similar socio-economic settings.

The study emphasizes the importance of taking care of the health and security dimension indicators for a better aging situation. The government of Bangladesh has begun giving old age allowances (though not to everyone), and has recently developed a draft of the ‘Parental Maintenance Rules, 2020’ [39]. To adopt the integrated active aging package (IAPP), Bangladesh requires a significant increase in the health and security dimensions of the active aging index. Implementing WHO models from primary data in Bangladesh was a challenge because it necessitated adapting all the questions suggested in the WHO aging framework into a Bangladesh context. Because the concept is so complicated, and there were time and resource limitations, some issues might be left out in this study. Some indicators had to be calculated based on the respondents' self-reported responses. As this is a cross-sectional study, it presented most of the self-reported responses of a large number of respondents. Therefore, the study findings may not be applicable for generalizing over the whole country. Given potential culture-oriented approaches in future studies, the calculated AAI construction responses of the respondents require further development, primarily in the psychological constructs related to the ability to cope with aging mechanisms, notably for the very old [40].

## 6 Conclusion

An analysis of the active aging situation in Bangladesh reveals that the AAI is not at a satisfactory level. The study findings cover the understanding regarding the wide range of indicators in the AAI, and existing elderly initiatives on these grounds have also been linked to finding the gaps and challenges. Respondents reported their understanding of the 18 indicators for the calculation of the AAI. In the health dimension of the AAI, the perceptions regarding crucial aspects of elderly health like chronic illness, health risk behaviors, and physical exercise were examined. Perceptions regarding the indicators for the participation dimension are very contextual in terms of the changing social context. The study also deduced how to interpret the security dimension indicators. These 18 indicators can aid in integrating the elderly into society and the economy. The findings are consistent with those of previous research on the subject. The conclusions of the study on the state of the health, participation, and security aspects of active adults are congruent with those of other studies in this field. Gender and residence-based variations were explored. Policymakers, researchers, and development partners will have a better understanding of the country's AAI status based on the findings. Thus, policymakers and researchers will know where to focus the attention and measures to enhance Bangladesh's active aging conditions in the present and future based on the researched obstacles and possibilities related to active aging. Relevant ministries will be able to take effective steps to restructure' programs in order to improve the state of affairs in these domains. Researchers can use the AAI to duplicate this tailored strategy for countries with similar socio-economic circumstances. Considering the future aging situation, the study findings will help allow for suitable programs to fulfill health needs, promote social participation and longer working lives, arrange education programs, and improve economic security to increase the level of active aging. Thus, opportunities for community participation should be made available for older adults in Bangladesh by creating an enabling environment for income generation and recreational facilities with age-specific plans and programs. These may include a policy extension of the retirement age, incentives for older workers, and incentives for employers employing older adults. Based on these findings, one can effectively plan health sector programs for older people. Healthy behavior can be promoted through cost-effective awareness programs. Immediate initiatives are needed to include the indicators of the AAI in different surveys of the Bangladesh Bureau of Statistics (BBS), so that the country can prepare an AAI and compare the progress of active aging every year. The systematic collection of such information on the indicators and domains of the AAI will allow scientists, policymakers, and researchers to formulate and implement strategies for improving the active aging situation.

## Declarations

### Author contribution statement

Md Aminul Haque: Conceived and designed the experiments; Performed the experiments; Analyzed and interpreted the data; Contributed reagents, materials, analysis tools or data; Wrote the paper.

Sadiya Afrin: Conceived and designed the experiments; Analyzed and interpreted the data; Contributed reagents, materials, analysis tools or data; Wrote the paper.

### Funding statement

This research did not receive any specific grant from funding agencies in the public, commercial, or not-for-profit sectors.

### Data availability statement

Data will be made available on request.

### Declaration of interest’s statement

The authors declare no conflict of interest.

### Additional information

No additional information is available for this paper.

## Appendix A Supplementary data

Questionnaire Sadiya Thesis Questionnaire Sadiya Thesis
