# Supplementary material for: Construction of the active aging index in Bangladesh: challenges and opportunities
Source: Heliyon. 2022 Oct 2;8(10):e10922. doi: 10.1016/j.heliyon.2022.e10922 (PMC9547210; doi:10.1016/j.heliyon.2022.e10922)
Supplement: Questionnaire Sadiya Thesis [file mmc1.docx]

**Questionnaire**

**“Perceptions, Determinants and Policy Issues of Active Ageing: A Study in Bangladesh”**

**Department of Population Sciences**

**University of Dhaka**

My name is ……………………………………………………, doing a thesis at Department of population Sciences, University of Dhaka. As part of the thesis, we are interviewing the elderly people from Dhaka and Rangpur Division. The study aims to explore the Perceptions, Determinants and Policy Issues of Active Ageing in Bangladesh.

**Confidentiality and Consent**

I would like to ask you some questions regarding my study objectives to answer. Your answers are completely confidential, your name will not be disclosed to anyone and will never be used with any of the information you give me. You do not have to answer any question that you do not feel comfortable. You may also withdraw from this interview at any time you want to. However, your answers to these questions will help us to understand the active ageing among elderly citizens. We would greatly appreciate your help in responding to this interview. Would you be willing to participate?

**IDENTIFICATION:**

| **SERIAL NUMBER** |  |
| --- | --- |
| NAME OF THE RESPONDENTS |  |
| ADDRESS OF The RESPONDENTS |  |
| CONTACT NO OF THE RESPONDENTs |  |
| NAME OF DATA COLLECTOR |  |
| DATE AND TIME |  |

**Questionnaire**

| **SECTION I: Background** | | | |
| --- | --- | --- | --- |
| Q. No. | Questions and filters | Options | Skip |
| 101 | What is your age? | ……………..Years |  |
| 102 | What is your Sex? | 1=Male  2= Female |  |
| 103 | Your current marital status? | Currently married  Separated  Widowed  Divorced  Never married |  |
| 104 | Are you head of the household? | 1=Yes  2=No |  |
| 105 | Have you ever studied? | 1=Yes  2=No | 107 |
| 106 | If yes, what is your years of schooling? | ………………………Years |  |
| 107 | What is your religion? | 1.Muslim  2.Hindu  3.Christian  4.Others |  |
| 108 | What is the total number your family? | ……………………… |  |
| 109 | What is the total number of children of you? | ………………………. |  |
| 110 | What is your family income? | ……………………………BDT |  |
| 111 | Do you have adequate sleep? | 1=Yes  2=No |  |
| 112 | How long can you sleep? | ……………………….hours |  |
| 113 | Do you walk in morning every day? | 1=Yes  2=No |  |
| 114 | Do you think that you get proper balanced diet? | 1=Yes  2=No |  |

| 115 | Do you use any Information and Communication technology? | 1=Yes  2=No | 117 |
| --- | --- | --- | --- |
| 116 | If yes, then what type of technology do you use? | 1=Mobile  2=Telephone  3=Computer  4=Others |  |
| 117 | Do you spend leisure in following ways? | 1=Yes  2=No |  |
|  | Watch TV | 1=Yes  2=No |  |
|  | Listen Radio | 1=Yes  2=No |  |
|  | Read Newspaper | 1=Yes  2=No |  |
| 118 | Can you make decision about following issues? | 1=Yes  2=No |  |
|  | Decision about study, marriage, job of children, grandchildren | 1=Yes  2=No |  |
|  | Decision about land buying or selling | 1=Yes  2=No |  |
|  | Decision about religious activities of family | 1=Yes  2=No |  |
|  | Decision about health care of family | 1=Yes  2=No |  |
| 119 | Do you think your house is elderly friendly? | 1=Yes  2=No |  |
| 120 | How much are you satisfied with your life | 1=Dissatisfied  2= Neither satisfied nor dissatisfied  3= Satisfied |  |

| **Section II: Elderly Health Related** | | | |
| --- | --- | --- | --- |
| Q. No. | Questions and filters | Options | Skip |
| 201 | What is your self-rated health status? | 1=Bad  2=Moderate  3=Good |  |
| 202 | What is your self-rated mental health status? | 1=Bad  2=Moderate  3=Good |  |
| 203 | Do you have any following disability? | 1=Yes  2=No |  |
|  | Paralysis | 1=Yes  2=No |  |
|  | Blindness | 1=Yes  2=No |  |
|  | Deafness | 1=Yes  2=No |  |

| 204 | Do you have taken the treatment of following diseases/ Doctor assured your following diseases? | 1=Yes  2=No |  |
| --- | --- | --- | --- |
|  | Arthritis | 1=Yes  2=No |  |
|  | High pressure | 1=Yes  2=No |  |
|  | Diabetes | 1=Yes  2=No |  |
|  | Cataract in last years | 1=Yes  2=No |  |
|  | Asthma | 1=Yes  2=No |  |
|  | COPD | 1=Yes  2=No |  |
|  | Stroke | 1=Yes  2=No |  |
|  | Heart attack | 1=Yes  2=No |  |
| 204.1 | Do you take medicine at regular basis? | 1=Yes  2=No |  |
| 204.2 | Do you think that there are health care services availability in your locality? | 1=Yes  2=No |  |
| 204.3 | Do you think that you can take health care services in need? | 1=Yes  2=No |  |
| 205 | Do you face any problem for doing the following daily activities? | 1=Yes  2=No |  |
|  | Bathing | 1=Yes  2=No |  |
|  | Dressing | 1=Yes  2=No |  |
|  | Eating | 1=Yes  2=No |  |
|  | Moving | 1=Yes  2=No |  |
|  | Toilet | 1=Yes  2=No |  |
| 206 | Do you face any problem for doing the following instrumental activities? | 1=Yes  2=No |  |
|  | Cooking | 1=Yes  2=No |  |
|  | Using mobile/telephone | 1=Yes  2=No |  |
|  | Shopping | 1=Yes  2=No |  |
|  | Uplifting 5 kg weight | 1=Yes  2=No |  |
|  | Housing look after | 1=Yes  2=No |  |
|  | Walking 1 km | 1=Yes  2=No |  |
|  | Crossing 2-3 stairs | 1=Yes  2=No |  |
| 207 | Do you have following bad habit for regularly? | 1=Yes  2=No |  |
|  | Smoking/drinking | 1=Yes  2=No |  |
|  | Chewing tobacco, zorda etc. | 1=Yes  2=No |  |

| **SECTION III: Elderly Participation Related** | | | |
| --- | --- | --- | --- |
| **Q. No.** | **Questions and filters** | **Options** | **Skip** |
| 301 | Are you engaged with any work beyond household work? | 1=Yes  2=No |  |
| 302 | Can you help in following household chores? | 1=Yes  2=No |  |
|  | Cleaning house | 1=Yes  2=No |  |
|  | Help in cooking | 1=Yes  2=No |  |
|  | Taking care of grandchildren | 1=Yes  2=No |  |
|  | Taking care of ill family member | 1=Yes  2=No |  |
|  | Helping in agriculture | 1=Yes  2=No |  |
|  | Washing clothes | 1=Yes  2=No |  |
|  | Looking after cattle | 1=Yes  2=No |  |
|  | Other works | 1=Yes  2=No |  |
| 303 | Are you engaged with any social/ religious group or religious institution |  |  |
|  | Old age group | 1=Yes  2=No |  |
|  | Religious group | 1=Yes  2=No |  |
|  | Voluntary social worker group | 1=Yes  2=No |  |
| 304 | Are you satisfied with family services? | 1=Yes  2=No |  |

| **SECTION IV: Elderly security related** | | | |
| --- | --- | --- | --- |
| **Q. No.** | **Questions and filters** | **Options** | **Skip** |
| 401 | What is your monthly income? | ……………….BDT |  |
| 402 | Is your monthly income is sufficient to fulfill your needs? | 0=no income  1=Not sufficient  2=Sufficient |  |
| 403 | What is the source of your income? | 1=Work  2=Pension  3=Old age allowance  4=Savings  5= Spouse  6=Children  7=relatives  8=Others |  |
| 404 | With whom are you living? | 1=Alone  2=Spouse  3=Son  4=Daughter  5= All in family  6=Other relatives |  |
| 405 | Is the house you reside is yours? | 1=Yes  2=No |  |
| 406 | Do you think your following housing environment are elderly friendly? | 1=Yes  2=No |  |
|  | Having separate room | 1=Yes  2=No |  |
|  | Bathroom facilities | 1=Yes  2=No |  |
|  | Toilet facilities | 1=Yes  2=No |  |
|  | Falls in old age | 1=Yes  2=No |  |
| 407 | Do you feel fear to when you are alone in house | 1=Yes  2=No |  |

| **SECTION V: Elderly perceptions regarding active ageing issues** | | | |
| --- | --- | --- | --- |
| **Q. No.** | **Questions and filters** | **Options** | **Skip** |
| 501 | What is the opinion of the elderly regarding the following active ageing issues | 1=Important  2=Not at all important/not important  3=Don’t know |  |
| 502 | What is the influence of not to have chronic illness for active ageing? | 1=Important  2=Not at all important/not important  3=Don’t know |  |
| 503 | What is the influence of having the ability to perform daily activities for active ageing? | 1=Important  2=Not at all important/not important  3=Don’t know |  |
| 504 | What is the influence of not to be suffering from health hazards for active ageing | 1=Important  2=Not at all important/not important  3=Don’t know |  |
| 505 | What is the influence of having no mental pain/depression for active ageing? | 1=Important  2=Not at all important/not important  3=Don’t know |  |
| 506 | What is the influence of avoiding health risk attitude for active ageing? | 1=Important  2=Not at all important/not important  3=Don’t know |  |
| 507 | What is the influence of having proper health services for active ageing | 1=Important  2=Not at all important/not important  3=Don’t know |  |
| 508 | What is the influence of economic work engagement for active ageing | 1=Important  2=Not at all important/not important  3=Don’t know |  |
| 509 | What is the influence of social work engagement for active ageing? | 1=Important  2=Not at all important/not important  3=Don’t know |  |
| 510 | What is the influence of intimate relations with family, friends for active ageing? | 1=Important  2=Not at all important/not important  3=Don’t know |  |
| 511 | What is the influence of having economic safety for active ageing | 1=Important  2=Not at all important/not important  3=Don’t know |  |
| 512 | What is the influence of being satisfied with housing for active ageing | 1=Important  2=Not at all important/not important  3=Don’t know |  |
| 513 | What is the influence of staying safe at home with housing for active ageing? | 1=Important  2=Not at all important/not important  3=Don’t know |  |
| 514 | According to you, which three factors are highly influential to ensure active ageing? | 1=Important  2=Not at all important/not important  3=Don’t know |  |
